# Investigation of the Role of Body Shape on an Air Lubrication System Inspired by Penguins

**DOI:** 10.3390/biomimetics11070501

**Published:** 2026-07-16

**Authors:** Arturo Giacobbe, Konstantinos Georgoussis, Giovanni Bianchi, Simone Cinquemani

**Affiliations:** Dipartimento di Meccanica, Politecnico di Milano, Via La Masa 1, 20156 Milano, Italy; giovanni.bianchi@polimi.it (G.B.); simone.cinquemani@polimi.it (S.C.)

**Keywords:** air lubrication, drag reduction, bioinspired engineering, penguin, torpedo

## Abstract

Bubble-assisted drag reduction is a promising strategy for improving the energy efficiency of underwater bodies, but its effectiveness depends not only on air injection but also on the ability of the body geometry to retain bubbles close to the surface. Drawing inspiration from the air retention and bubble release mechanisms of penguin plumage, this study examines whether a simplified penguin-inspired geometry can enhance bubble coverage compared to a conventional axisymmetric body. Prototypes featuring a penguin-inspired shape and a torpedo shape, each equipped with an air diffuser and injection holes, were designed and evaluated in a dedicated towing tank. At low air pressure, the penguin-inspired body demonstrated a 31.5% reduction in drag coefficient compared to the condition without bubbles. In contrast, the torpedo-shaped body did not exhibit a reduction in drag coefficient under identical air-injection conditions. Both geometries showed diminished performance at higher pressures, likely due to bubble growth, coalescence, and decreased near-wall retention. These findings indicate that the effectiveness of bubble-assisted drag reduction is highly dependent on body geometry and that bioinspired body morphology may play a critical role in sustaining a layer rich in air bubbles near the surface. This study offers a preliminary experimental assessment of penguin-inspired air lubrication and identifies bubble size, air-flow control, and quantification of surface coverage as key areas for future research.

## 1. Introduction

Reducing hydrodynamic drag remains a primary challenge in the development of efficient underwater systems, such as marine vessels, autonomous underwater vehicles, and bio-inspired aquatic robots. In maritime transportation, reducing drag directly decreases fuel consumption and emissions [1,2]. For small autonomous platforms, it extends operational endurance and enhances mission capability [3,4,5,6,7,8]. Consequently, a wide range of passive and active drag-reduction strategies have been investigated, including surface coatings, compliant or textured surfaces, and air-based lubrication methods [5,6].

Among these approaches, air lubrication is particularly attractive because substituting part of the liquid–solid interface with a gas-rich layer reduces the effective shear stress on the body surface. However, the performance of air-lubrication systems depends on several interrelated parameters, such as bubble size, air injection rate, local flow conditions, and the capacity of injected air to remain near the surface instead of dispersing into the surrounding flow.

Biological swimmers provide useful examples of how morphology and surface properties can interact with the surrounding fluid. Penguins are particularly relevant in this context because their plumage traps air during diving and releases it during rapid underwater motion [9]. This phenomenon is associated with the formation of an air-rich region around the body, which may contribute to drag reduction during rapid ascent and porpoising motions [9,10].

The plumage architecture of penguins, consisting of overlapping feathers and a highly porous down layer, supports air retention and has also inspired studies on thermal insulation, anti-icing, and fluid-interaction mechanisms [10,11,12]. Although the biological system is considerably more complex than an engineered prototype, it suggests that drag reduction through air bubble release is not only about injecting bubbles, but also about maintaining them near the body surface.

Most engineering studies on air lubrication focus on the generation of bubbles or air layers and on their influence on the global drag of simplified bodies or ship-like configurations [13,14,15,16]. Less attention has been devoted to the role of body morphology in promoting or inhibiting bubble retention. This aspect is particularly relevant for bioinspired underwater vehicles, where the external shape may influence whether injected bubbles form a surface-attached layer or detach rapidly from the body. Therefore, an open question is whether a simplified penguin-inspired geometry can improve bubble coverage and drag reduction compared with a conventional streamlined geometry when the same bubble-generation system is used.

The present work addresses this question by designing and experimentally characterizing a penguin-inspired air-lubrication system. Two underwater bodies were fabricated and tested under comparable conditions: a flattened penguin-inspired prototype and a torpedo-shaped reference body. The two models were designed with the same length and maximum frontal area, but differed in overall morphology. Both bodies were equipped with a porous diffuser and air-injection holes near the upstream region, so that the injected bubbles could interact with the external flow along the downstream surface. A dedicated towing-tank test bench was developed to measure the drag force acting on the prototypes while they were pulled through water. The velocity of the bodies was estimated using video-based tracking against a calibrated checkerboard background, while the drag force was measured with a load cell. In addition, image-processing algorithms were used to estimate bubble count, bubble-size distribution, and qualitative surface coverage at different air-supply pressures. The objective was not to replicate the full complexity of penguin plumage, but to isolate a simplified biomimetic principle: the possible role of body geometry in retaining injected air bubbles near the surface.

The main contribution of this study is an experimental comparison showing that the same air-injection concept can lead to different drag outcomes depending on body shape. At low air-supply pressure, the penguin-inspired body showed a reduction in drag coefficient, whereas the torpedo-shaped body did not benefit from bubble injection. At higher pressure, performance deteriorated, suggesting that excessive air injection may promote the formation of larger bubbles, coalescence, and detachment from the surface. These findings support the hypothesis that bubble-assisted drag reduction is governed by the combined effect of air injection and geometry-dependent bubble retention, and they provide design indications for future bio-inspired underwater vehicles.

## 2. Air Retention in Penguin Plumage and Engineering Scaling

The biological inspiration for the present work is the air-retention and air-release capability of penguin plumage during underwater motion. Penguin plumage is not a simple external covering, but a dense hierarchical structure composed of different feather types with distinct functions. In emperor penguins, stiff contour feathers form the outer waterproof layer, while afterfeathers and plumules form the downy insulating layer beneath the contour feathers [17]. Williams et al. showed that plumules had been overlooked in previous descriptions and models of penguin insulation, and reported that plumules are approximately four times as numerous as afterfeathers. Filoplumes are also present and are likely associated with sensory functions, possibly helping the animal detect displaced contour feathers and maintain a smooth external plumage arrangement. The unique arrangement places nine plumules surrounding each contour feather in a complex, non-uniform matrix. This dense, overlapping mat of downy feathers forms a structure that physically traps and retains up to 5 liters of air [18,19]. It has been observed that, in emperor penguins, the mean distance between adjacent contour feathers is 1.75 mm, with an average feather length of 24 mm. Feather density typically ranges from 5.8 to 10.5 feathers/cm^2^ on the dorsal region and from 8.7 to 13.5 feathers/cm^2^ on the ventral region [9,17]. Keeping the feathers tightly packed over the body enhances the microscopic air–water separation, forming a continuous layer through which water cannot penetrate. During a rapid underwater ascent, the air trapped within this dense downy layer expands and is intentionally released into the bird’s boundary layer. The high density of the plumules combined with their specialized barbule structure breaks down the escaping air into an extremely fine distribution of micro-bubbles that surround the penguin’s body [20]. This process reduces hydrodynamic drag, acting as a lubricant that allows the penguin to reach higher underwater speeds necessary to propel itself out of the water.

During the rapid descent and air ejection process, the feathers are subjected to several external forces, and this might cause the sudden detachment of a feather from the penguin’s body. If air or water flow disrupts a contour feather, it alters the local pressure field and deflects the tip of the adjacent filoplume [21]. The deflection acts on a mechanoreceptor at the feather’s base, sending a signal that alerts the penguin exactly where it needs to preen to restore the airtight, waterproof seal. This complex feather architecture is relevant to the present study because it provides both a porous air-retaining volume and an external morphology that can influence how air is released into the surrounding flow.

The air–water separation mechanism is based on the inner nanostructure of a penguin’s feathers and on their surface tension. Similarly to other birds, feathers of penguins are composed of wire-like barbs and barbules that form a rough feather surface [22,23]. The interwoven mesh formed by feather barbs and barbules leaves small pores whose size is estimated to be less than 20 μm. An additional layer of preen oil is distributed over the feather’s surface. This augments water shedding by coating nanoscale grooves and making them more hydrophobic [24]. Overall, the combination of the nanoscale structure and the oily surface covering allows penguins to achieve a Cassie–Baxter wetting state [25], effectively separating outer water from the inner entrapped air [26,27]. In this particular state, water sits partly on feather solids and partly on trapped air within the barbule network rather than fully penetrating it [28,29].

Once the air–water separation effects of single feathers is combined, the plumage can be modeled as a uniform porous layer that surrounds the body of the penguin. The porosity of penguin feathers and down is particularly important for air storage. Du et al. developed a heat-transfer model for Gentoo penguin feathers and down and estimated a mean porosity of 96.1% for the feather assembly, using a representative feather thickness of 24 mm [30]. In their model, the barbules were represented as fine fibers with a radius of approximately 3 μm, and the authors concluded that both fiber fineness and geometrical arrangement contribute to the thermal performance of the plumage. Although that study focused on heat transfer rather than hydrodynamic drag, it supports the view that penguin plumage can act as a highly porous, air-rich medium.

Additional evidence of air retention is provided by studies on penguin feather wettability and anti-icing behavior. Wood et al. showed that the body feathers of the Gentoo penguin (Pygoscelis papua) are arranged in an overlapping pattern and that their water-shedding behavior is associated with air trapping within a wire-like microstructure [31]. At the microscale, the feather pinnae include barbs with diameters of approximately 30–35 μm, branching barbules with diameters of approximately 10–15 μm, and hooked hamuli with diameters of approximately 2–5 μm. This hierarchical morphology promotes air entrapment and provides a useful biological analogue for engineered surfaces intended to maintain air close to a submerged body.

The possible hydrodynamic role of this trapped air was formulated by Davenport et al. as an air-lubrication hypothesis for jumping emperor penguins [9]. From underwater video analysis, they reported that rapidly ascending emperor penguins produced long bubble wakes and reached mean ascent speeds of approximately 5.3 m s^−1^, considerably higher than their horizontal and descending swimming speeds. They also reported that fine bubbles emerged from the plumage over much of the body, forming a tight bubble cloud around the animal before being convected into the wake. The proposed mechanism is that penguins load air into their plumage at the surface, dive to depths of approximately 15–20 m, and subsequently release compressed air during ascent as the hydrostatic pressure decreases. The release of air into the boundary layer may reduce the drag force by forming an air-rich region close to the body surface that modifies the effective mixture properties and the turbulent momentum transport close to the body surface. However, this mechanism involves several coupled effects, including feather deformation, air compression and expansion, body motion, buoyancy, surface chemistry, and near-wall multiphase flow, which make this strategy effective only under precise conditions of flow velocity, body geometry, and bubble size and distribution.

In engineering terms, air lubrication can occur in different regimes. At relatively low gas flux, the injected air remains dispersed as bubbles within the near-wall flow, a regime commonly referred to as Bubble Drag Reduction (BDR) or, when the bubbles are sufficiently small, MicroBubble Drag Reduction (MBDR). At higher gas flux, bubbles can coalesce into a more continuous air layer, leading to Air-Layer Drag Reduction (ALDR), while still larger and more stable gas structures may form air cavities [1,32,33]. The distinction between these regimes is important because they differ in required gas flow rate, persistence downstream of the injection point, and drag-reduction mechanism [33].

The present work is closer to the BDR regime than to a controlled ALDR or air-cavity system, because the prototype generates a dispersed bubble cloud rather than a stable continuous gas layer. The effectiveness of BDR depends strongly on the presence of gas within the near-wall region. Ceccio notes that drag reduction is strongly related to the near-wall void fraction, because the turbulent transport processes responsible for skin-friction drag occur within a small region close to the wall [32]. Several mechanisms can contribute to drag reduction: the gas modifies the effective density and viscosity of the mixture, bubbles interact with turbulent structures, the local mixture becomes more compressible, and bubble breakup and coalescence can alter the near-wall flow. This means that the total amount of injected air is not, by itself, a sufficient predictor of drag reduction, as bubbles must remain close to the surface over a sufficient downstream distance. If they migrate away from the wall, escape laterally, or coalesce into larger detached structures, the reduction in wall shear stress may be weakened and the total drag may increase.

This point is directly relevant to the biomimetic question addressed in this study, because the penguin-inspired prototype is not intended to reproduce the feather microstructure itself. Instead, it abstracts two features of the biological system: air release near the body surface and a non-axisymmetric body morphology that may favor bubble retention along the lower surface. The torpedo-shaped prototype, in contrast, represents a conventional streamlined axisymmetric geometry. By equipping both bodies with the same air-release concept and testing them under comparable towing conditions, the study evaluates whether body morphology affects bubble retention and the resulting drag response.

A first-order-of-magnitude comparison between the biological reference and the experimental prototype can be made using the Reynolds number,

(1) $$Re=\frac{\rho UL}{\mu },$$

where ρ is the water density, *U* is the characteristic velocity, *L* is the characteristic body length, and μ is the dynamic viscosity of water.

Bubble behavior in bubble-drag-reduction studies is commonly characterized using inner turbulent-boundary-layer scales, and two relevant parameters are the bubble diameter normalized by the viscous length, $d_{B}/l_{\nu }$, and the Weber number based on the friction velocity [32]

(2) $$We=\frac{\rho d_{B}u_{*}^{2}}{\sigma },$$

where $d_{B}$ is the characteristic bubble diameter, σ is the water–air surface tension, and $u_{*}$ is the friction velocity associated with the local wall shear stress $\tau_{w}$ and computed as follows:

(3) $$u_{*}=\sqrt{\frac{\tau_{w}}{\rho }}$$

Since $\tau_{w}$ was not measured locally in either the biological reference case or the present experiments, it was estimated from the expression of the shear stress within a turbulent boundary layer of a flow close to a flat surface [9,34].

(4) $$\tau_{w}≃\frac{1}{2}\rho U^{2}\times 0.074\;Re^{-1/5}$$

This estimate is used only for order-of-magnitude comparison, since neither the penguin body nor the present prototypes are flat plates and local curvature, pressure gradients, surface roughness, and bubble injection can modify the actual wall shear stress.

For the biological reference case, using the mean ascent velocity reported by Davenport et al. [9] for emperor penguins (aptenodytes forsteri), $U≃5.3\;m\;s^{-1}$, and a characteristic length L≃1m gives $Re∼5\times 10^{6}$ and $u_{*}≃0.22\;m\;s^{-1}$. Yoda and Ropert-Coudert [35] report a characteristic length of L≃0.4m for Adélie penguins (Pygoscelis adeliae) and velocity $U≃2\;m\;s^{-1}$, resulting in $Re∼8\times 10^{5}$ and $u_{*}≃0.098\;m\;s^{-1}$.

Since the diameter of bubbles released by natural plumage was not directly measured, a unique biological Weber number cannot be assigned. Assuming characteristic bubble diameters of 20μm, consistent with release through a fine porous plumage structure, gives $We∼10^{-2}$.

To assess how the bubble sizes generated in the experimental prototype compare with the characteristic scales of the near-wall turbulent flow, the ratio $d_{B}/l_{v}$ was evaluated as a function of bubble diameter, following the flat-plate approximation. The prototypes were characterized by a characteristic length L=0.293 m and a characteristic velocity of U=1.1 m s^−1^. Further explanations on the physical parameters of the prototypes are provided in the next sections. The Reynolds number was first computed from Equation (1), yielding $Re∼3\times 10^{5}$. This value was then substituted into the flat-plate wall-shear correlation (Equation (4)), giving an estimated wall shear stress of $\tau_{w}\approx 3.54$ Pa. The corresponding friction velocity was obtained from Equation (3), resulting in $u_{*}\approx 0.0595$ m s^−1^. The viscous wall length scale then follows directly as $l_{v}=\nu /u_{*}\approx 16.8\;\mu$m. Under this flat-plate estimate, $l_{v}$ depends only on the bulk flow parameters (U,L,ν) through $\tau_{w}$, and is therefore independent of the bubble diameter $d_{B}$. Consequently, $l_{v}$ is not itself a function of $d_{B}$; rather, it acts as a single length-scale constant against which bubble sizes can be normalized.

**Figure 1:**
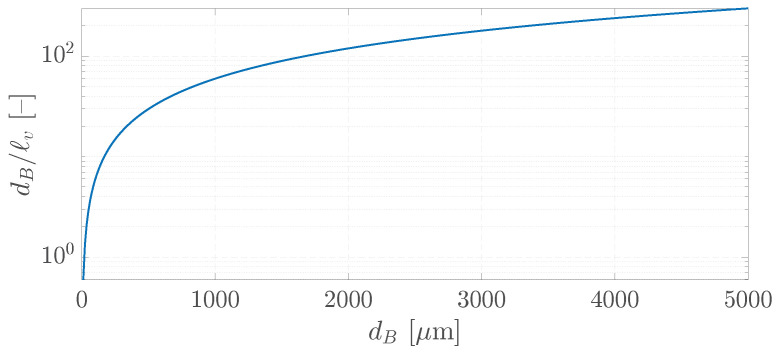
Dimensional analysis of the bubble’s characteristic scale for increasing $d_{B}$. Bubble diameter goes from a minimum value of 10μm up to the average value of 5000μm observed later in the tests with the prototypes.

A range of bubble diameters $d_{B}\in \left[20,5000\right]\;\mu$m was sampled, covering the interval from the minimum pore size used in literature [18,30] to the average bubble diameter found in the experiments of the present study. For each value of $d_{B}$, the normalized diameter was computed as the ratio $d_{B}/l_{v}$, using the value of $l_{v}$ obtained above. Because $l_{v}$ is constant over the sweep, this operation reduces to a linear rescaling of $d_{B}$ by the constant factor $1/l_{v}\approx 0.0595\;\mu$m^−1^. Figure 1 shows the normalized characteristic length for varying bubble diameter.

In penguins, air retention and release depend on feather-scale morphology, plumage deformability, hydrophobic surface chemistry, hydrostatic compression during diving, body motion, and possibly active feather control. Reproducing all these features would make it difficult to identify which aspect of the system is responsible for the observed drag response. For this reason, the present study adopts a reduced biomimetic approach: the feather-scale air-storage mechanism is replaced by a controlled air-release system, while the biological body morphology is represented by a simplified penguin-inspired geometry.

This simplification is appropriate for the objective of the study, which is not to reproduce the complete penguin air-lubrication mechanism, but to test whether body geometry affects the ability of injected bubbles to remain close to the surface. Accordingly, larger manufactured holes and a 3D-printed body are acceptable as a first-order engineering abstraction, even though they generate bubbles that are much larger than those expected from natural plumage. Using the formula suggested by S.H. Marshall [36] for bubble size in cross-sectional flow, the bubble radius emitted by the penguin can be computed as

(5) $$R_{b}=0.48\;R_{o}^{0.826}{\left(\frac{U_{gi}}{U_{L}}\right)}^{0.36}$$

where $R_{b}$ is the volume-equivalent bubble departure radius, $R_{o}$ is the orifice radius, $U_{gi}$ is the superficial orifice gas velocity, and $U_{L}$ is the mean liquid cross-flow velocity. The mean bubble radius is calculated to be 3.43 μm. The resulting prototype should therefore be interpreted as a comparative model for studying geometry-dependent bubble retention, not as a dynamically scaled replica of penguin plumage.

Table 1 summarizes the biological features considered, their simplified engineering translation, and the main limitations introduced by this abstraction.

## 3. Materials and Methods

### 3.1. Test Bench Design

**Figure 2:**
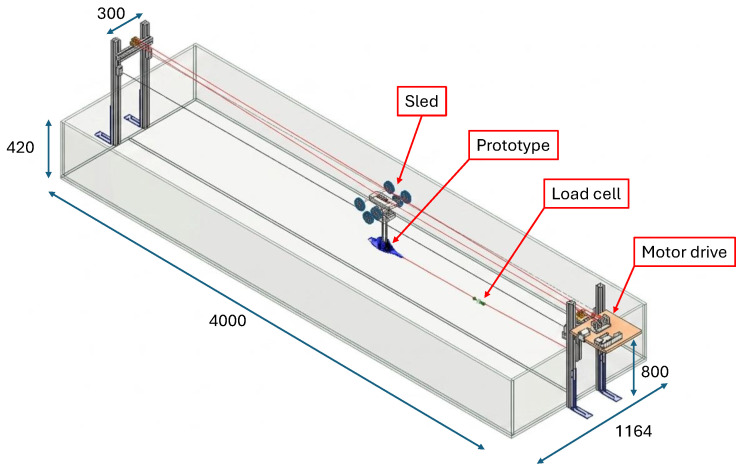
The experimental test bench is a towing tank with a double-tower structure and a pulling mechanism inspired by the one used in aerial cable cars. The different components are labeled, together with dimensions in millimeters.

The experimental test bench consists of a towing tank specifically designed to accommodate the underwater vessel, the towing mechanism, and the sensors and cameras required for the experiments. The system uses a structure to support the tensioned cables, which are attached to two towers positioned on each side of the water tank and equipped with a pulley system connected to a DC motor that can pull the vessel in both directions. The vessel is mounted beneath the sled using four threaded bars, and a load cell is attached to measure the total drag force. Figure 2 illustrates the test bench design and its dimensions.

**Figure 3:**
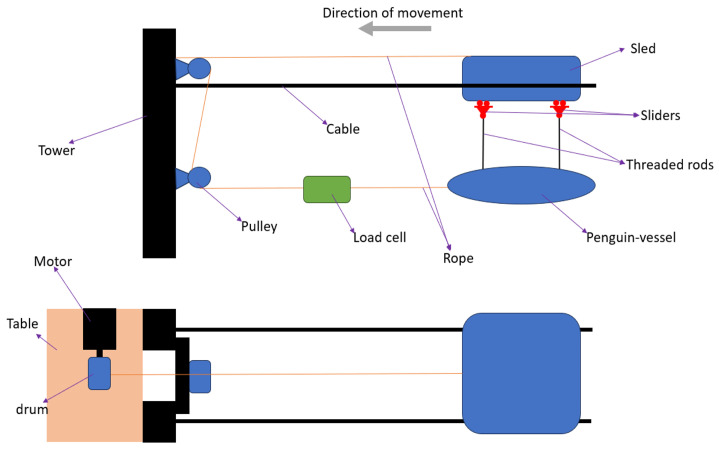
The towing mechanism consists of two supporting cables (black), plus one central cable (orange) for pulling the sled. The central cable is connected to a motor that pulls the entire mechanism.

Figure 3 shows the system for constraining the body using a sled and tensioned cables. The underwater body is rigidly constrained to the sled with four bars, and the sled is supported by two tensioned cables to prevent unwanted transversal displacements and rotations, so the vessel’s only degree of freedom is horizontal displacement. The cables are tensioned using two adjustable external springs, which secure the sled and enhance its resistance to external disturbances. An additional cable is attached at the center of the sled, enabling the DC motor to pull both the sled and the vessel.

**Figure 4:**
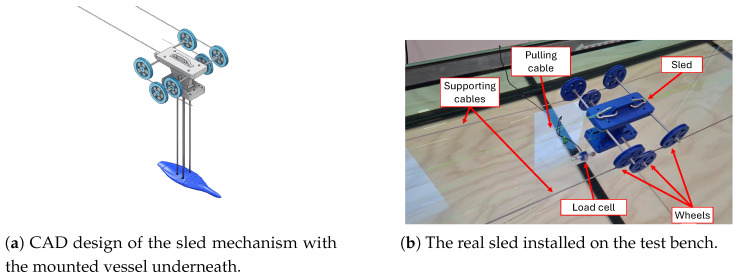
Sled moving on supporting cables thanks to a series of slotted wheels. All the dimensions are in millimeters.

The sled moves along the supporting cables using a series of slotted wheels. Each wheel features a groove that encloses the cable, thereby enhancing system stability. Three wheels are mounted on each supporting cable: two positioned above to facilitate a smooth rolling, and one positioned below to grip the cable and ensure pure rolling without sliding. Figure 4 shows the mounted wheels and the prototype under the sled.

The primary objective of this study is to measure the total drag force acting on the underwater body during motion. To avoid including friction forces from the towing mechanism, the underwater vessel is decoupled from the towing system by permitting relative horizontal motion between the body and the sled. This configuration enables the system to reach a steady-state equilibrium without external constraints, resulting in an accurate measurement of the drag force.

Additional components required for system operation include a DC motor, a motor drive, a potentiometer, an Arduino Due, and a power supply. The motor is a brushed DC motor (24 V, 3500 rpm) RS555 Series (Mellor Electric Blackburn, Blackburn, UK), with a maximum output torque of 10 Ncm, and the motor drive used is an L298N board. The drum connected to the motor, which retrieves the rope, has a radius of 10 mm. This configuration produces a maximum pulling force of 10 N on the underwater prototype, enabling the vessel to reach a maximum speed of 1.11 m s^−1^.

Prior to the experimental tests, the vessel was mounted on the sled, a load cell was attached upstream of the vessel, and a camera was positioned in front of a calibration checkerboard to record the test. The sled was then pulled by the DC motor connected to the drum. Tests were conducted both with and without bubble emissions for the penguin-shaped and torpedo-shaped vessels, and at different air supply pressures.

In these experiments, the prototypes have a length of L=0.293 m and achieve velocities up to approximately U=1.11 m s^−1^, corresponding to $Re∼3\times 10^{5}$ in water. The Reynolds numbers for the two prototypes examined in this study are comparable to those calculated by Yoda and Ropert-Coudert [35] for Adélie penguins.

### 3.2. Penguin-Inspired Vessel

Since the objective of this study is to understand which parameters are important to mimic the drag-reduction effect obtained by penguins, the vessel’s geometry was one of the investigated parameters, and two different prototypes were developed: one with a penguin-inspired shape and another with a shape more similar to conventional torpedoes.

**Figure 5:**
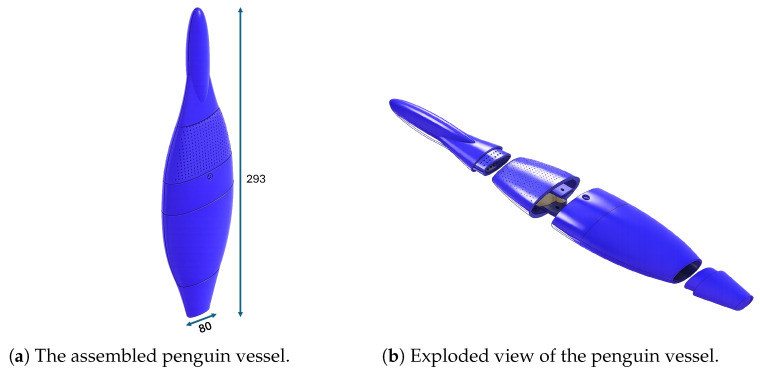
Design of the penguin vessel.All the dimensions are in millimeters.

The penguin shape was approximated using an elliptical cross-section, and the overall geometry is shown in Figure 5. The prototype was 3D-printed using PETG.

Penguins have a streamlined body, with a sharp beak and a torso that enlarges up to the midsection of the penguin. For emperor penguins, total height is around 100 cm but varies by individual, and the largest cross-sectional area is around 500 cm^2^. These values vary based on the individual and the season. This results in a cross-sectional radius of 12.62 cm. Assuming the penguin approximates an ovoid shape, the fineness ratio (body length to maximum body diameter) is 3.96. In the prototype developed, the fineness ratio is 3.67. The selected elliptical cross-section is flatter than that of the penguin to provide a shallower underside angle, thereby reducing the rapid ascent of bubbles as they exit the vessel.

Compared with a penguin’s morphology, the designed shape incorporates a beak-like anterior feature in both cases. Although the elliptical cross-section adopted for the vessel is more elongated and less circular than that of a natural penguin, this modification was introduced to enhance bubble attachment along the surface. A tail-like posterior feature was also included in the model. The vessel has a length of 29.3 cm and a maximum cross-sectional area of 18.84 cm^2^. An exploded view of the prototype is shown in Figure 5. The vessel features a double-ellipse profile in both the cross-sectional plane and the length direction. The second part has holes 0.6 mm in diameter through which air is released; these holes are positioned just before the largest cross-sectional area and near the front, so they can cover most of the surface while the robot is moving. This part also houses the porous stone, as shown in Figure 5, used to create very fine bubbles.

**Figure 6:**
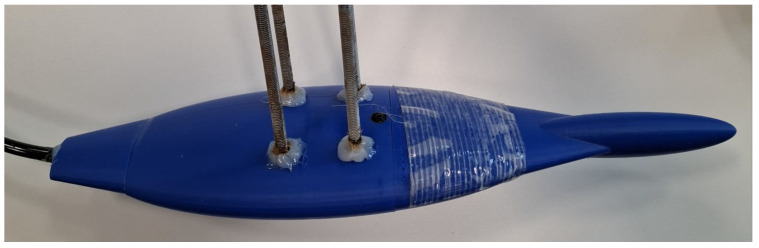
3D-printed penguin vessel with the threaded rods attached.

Figure 6 presents the 3D-printed model with threaded rods attached for mounting to the test bench. Adhesive was applied to most connections between components to ensure an airtight seal and prevent bubble leakage through unintended gaps.

### 3.3. Torpedo Shaped Vessel

To assess the influence of vessel shape on air lubrication, a generic torpedo-shaped vessel was designed. The entire prototype was fabricated using a 3D printer and PETG material.

**Figure 7:**
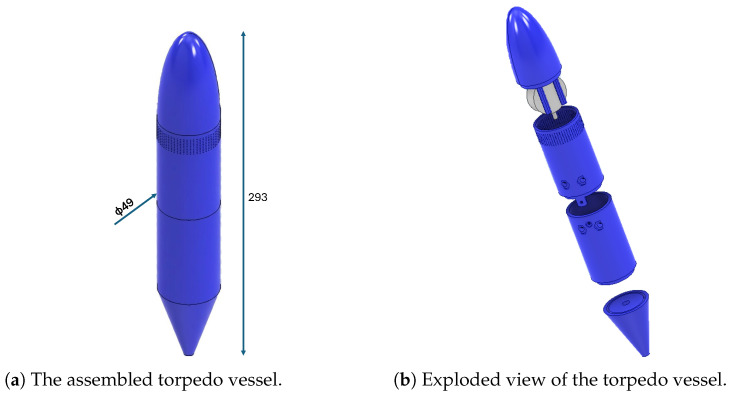
Design of the torpedo vessel. All the dimensions are in millimeters.

The vessel consists of four 3D-printed parts that are glued together to prevent air leakage during testing. The front section serves as the nozzle. At the rear, four bars extend outward to support the aquarium porous stone, which generates fine bubbles, as indicated by the gray component in Figure 7. The subsequent component contains 0.6 mm holes for air release and is positioned near the front of the vessel, after the cone, to ensure bubble coverage along most of the vessel. Both this part and the third section include holes for threaded rods, enabling attachment to the sled. The tail section features an opening for the plastic tube that connects to the air supply.

**Figure 8:**
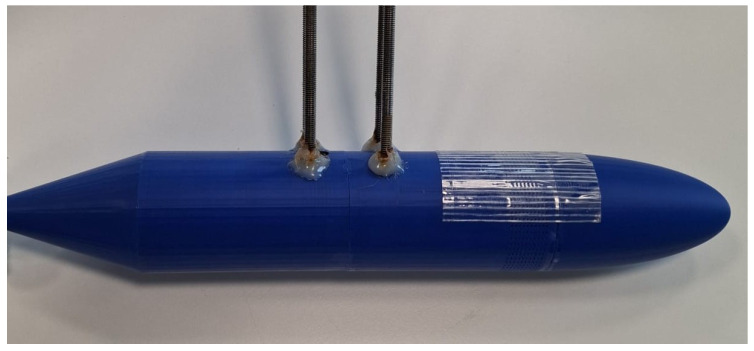
3D-printed torpedo vessel with the threaded rods attached.

As shown in Figure 8, the pieces have a good surface finish. The tolerances are also adjusted so that the cylinder is continuous without having edges where the components connect. The vessel is 29.3 cm long and has a maximum cross-sectional area of 18.84 cm^2^, the same as the penguin-inspired one.

Table 2 presents a comparison of the design parameters for both prototypes, including their external surface areas and additional details regarding the air ejection systems.

## 4. Experimental Testing and Results

This section details the experimental setup, data processing methodology, and results. The primary outcomes include the force measured with a load cell, body velocity, and bubble count and diameter, determined using computer vision algorithms. All the measurements were performed in steady-state conditions, once the vessels reached a constant velocity.

### 4.1. Experimental Testing

#### 4.1.1. Velocity Calculation

To determine vessel velocity, a checkerboard was positioned behind the tank, and a camera was placed in front of it. The checkerboard consisted of a white-and-red grid with 2 × 2 cm squares.

A computer vision algorithm was developed with MATLAB 2025b to measure the vessel’s speed as it traversed the grid. The script tracks the blue object and calculates its velocity, incorporating corrections for perspective distortion since the vessel is positioned closer to the camera than the background grid.

**Figure 9:**
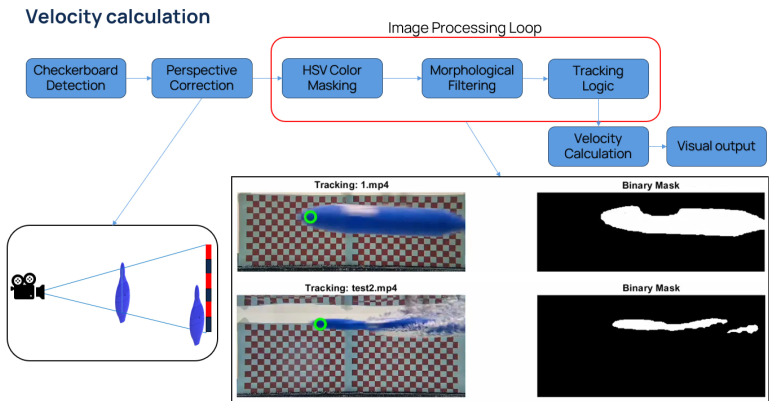
Block diagram for velocity tracking; the green circle shows the average of the first 100 pixels of the vessel.

The algorithm first reads the MP4 file containing the vessel’s motion and decodes it into individual frames. It then corrects for perspective, accounting for the camera’s position (120 cm from the grid and 60 cm from the object). The grid is identified in the background, enabling conversion from pixels to centimeters. The script utilizes the HSV (Hue, Saturation, Value) color space to detect the blue vessel, filtering for a specific hue range (0.55 to 0.75) corresponding to blue, and fills any holes in the detected object to ensure a solid shape. The blue filtering process is illustrated in Figure 9, where the binary mask displays detected blue regions in white and all other areas in black.

Rather than tracking the object’s center, the algorithm specifically identifies the front edge of the vessel. It detects the largest blue region, sorts the corresponding pixels by their X-coordinate, and averages the leftmost pixels to represent the vessel’s nose, as shown in Figure 9.

After recording the coordinates throughout the video, the algorithm calculates the vessel’s actual speed. It determines pixel displacement between frames using the Euclidean distance, converts this displacement to centimeters, applies the perspective correction factor, and multiplies by the frame rate to obtain velocity in centimeters per second. The formulation is presented below:

(6) $$V_{real}=\left(\sqrt{{\left(x_{t}-x_{t-1}\right)}^{2}+{\left(y_{t}-y_{t-1}\right)}^{2}}\cdot C_{px}\cdot \frac{d_{vessel}}{d_{grid}}\right)\cdot f_{s}$$

- $V_{real}$: Perspective-corrected velocity (cm/s);
- $\sqrt{{\left(x_{t}-x_{t-1}\right)}^{2}+{\left(y_{t}-y_{t-1}\right)}^{2}}$: Euclidean pixel displacement;
- $C_{px}$: Calibration factor (cm/px);
- $d_{vessel}$: Distance to vessel (60cm);
- $d_{grid}$: Distance to calibration grid (120cm);
- $f_{s}$: Frame rate (FPS).

A median filter is applied to eliminate sudden spikes in velocity resulting from tracking errors. Finally, the algorithm plots the velocity and provides a display to verify tracking accuracy.

#### 4.1.2. Force Measurements and Calculation of Drag Coefficient

**Figure 10:**
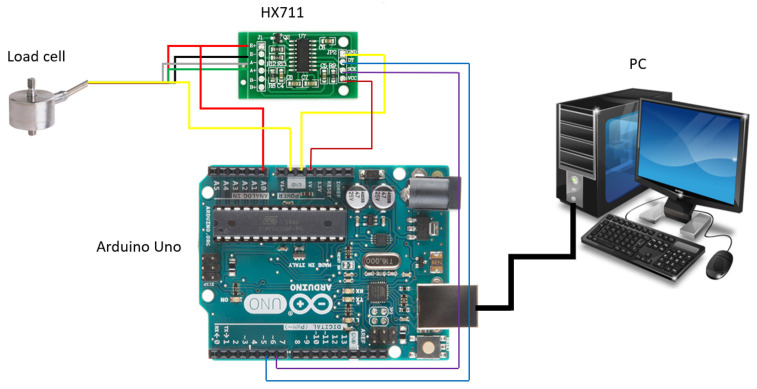
Schematic diagram of the connections of the load cell.

The force measurement was performed using a load cell CN1501541594 (XNQ Electric Company Store, Beijing, China). The load cell was calibrated by the producer; its sensitivity is 0.8 mV/V, and its full scale is ±30 N. The load cell was connected to an HX711 amplifier and ADC module and an Arduino Uno for acquiring the signal, as shown in Figure 10.

Custom Arduino code was developed to extract data from the load cell at 5-s acquisition intervals. Subsequently, a code was implemented in Python 3.12 to transfer data from the Arduino to a computer, read the appropriate port, and store test results in data files. A MATLAB script was then used to convert these data sheets into force-over-time diagrams. Utilizing the mean velocity calculation, the drag coefficient $c_{d}$ was also determined.

#### 4.1.3. Bubble Tracking

A MATLAB computer vision algorithm was implemented to track, count, and measure the size of bubbles exiting the vessel. Unlike the previous script, which tracked a single moving vessel, this algorithm is designed for Bubble Size Distribution (BSD) analysis. It detects multiple small objects, manages clumping, and calculates statistical diameters. The algorithm processes MP4 video files as input.

**Figure 11:**
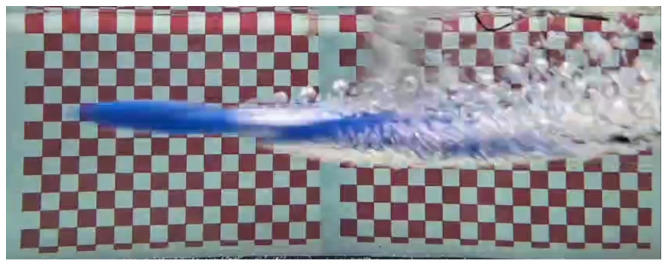
Frame from the video that is used for bubble tracking.

**Figure 12:**
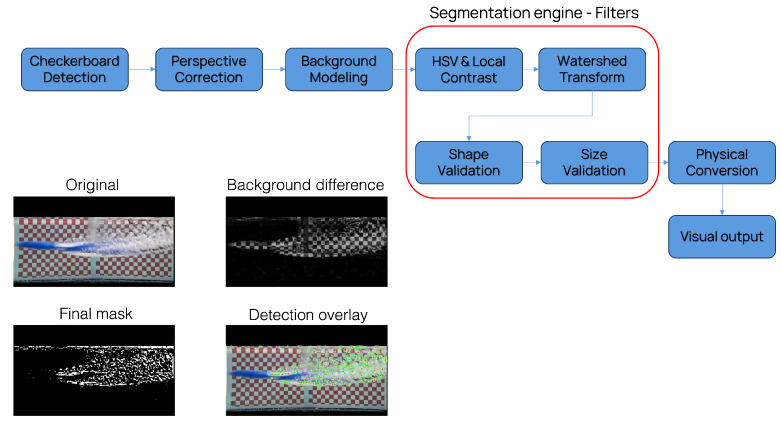
Block diagram from the algorithm and frame from the display window that is used for bubble tracking.

The initial frames of the input video are used to calibrate the dimensions from pixels to centimeters using a checkerboard background pattern. Figure 11 presents a screenshot from a video used for bubble tracking. This approach enables calculation of the bubbles’ physical dimensions. As in previous analyses, a perspective factor of 60/120=0.5 is applied, given that the checkerboard is 120 cm from the camera and the bubbles are located at 60 cm. After detection, the algorithm stores the first frame displaying only the checkerboard. During image pre-processing to isolate bubbles, three filters are employed to distinguish bubbles from the complex background. The first is an HSV color filter that selects regions with low saturation (S < 0.5), since bubbles are typically clear or white/gray and thus exhibit minimal color relative to the blue vessel. The second filter, local contrast enhancement, subtracts a blurred version of the image from the original, functioning as a high-pass filter to emphasize bubble edges while minimizing lighting variations. The final filter, motion masking, compares the current frame to the initial checkerboard frame, removing static background noise and reflections, as bubbles are in constant motion. Video analysis revealed frequent interactions among bubbles, including touching and overlapping (Figure 11). A basic detector would interpret two touching bubbles as a single large object. To address this, the algorithm employs a Watershed Transform, which calculates the distance from each white pixel to the nearest black pixel, designating the white pixel as the bubble center and peak. The results of this filtering process are presented in Figure 12, where the mask display demonstrates effective detection and analysis of bubble clusters.

To minimize the influence of random optical noise, the algorithm incorporates a quality-control shape filter. This filter excludes objects that lack sufficient roundness or exhibit excessive elongation, such as light reflections. Additionally, it disregards objects that are either too small or excessively large to further reduce background noise. Ultimately, the algorithm extracts metrics including mean bubble diameter, bubble count, and coefficient of variation from the resulting plots.

### 4.2. Results

#### 4.2.1. Velocity Results

The initial comparative analysis involved calculating the velocities of the vessels using the computer vision algorithm described in Section 4.1.3. For both the penguin-inspired and the torpedo prototypes, speed was measured under three conditions: no bubble activation and low-pressure (0.5 bar) and high-pressure (1.5 bar) bubble injection. The air flow rates for tests at 0.5 bar and 1.5 bar were 0.41 l/s and 0.59 l/s, respectively.

**Figure 13:**
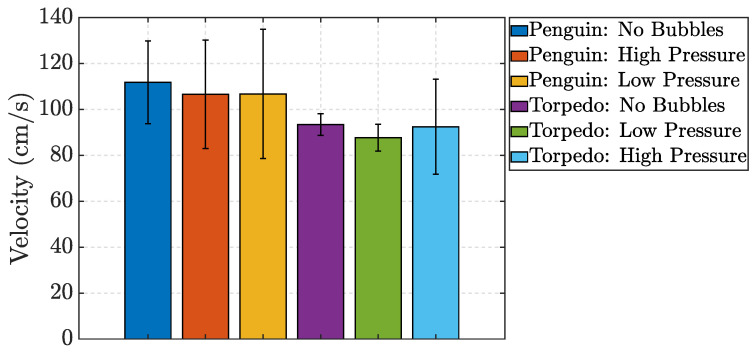
The mean velocities from all the tests for both vessels. The error bars correspond to ±σ.

Five tests were conducted for each vessel in each conditions and Figure 13 summarizes the mean velocities from each test.

#### 4.2.2. Force Results and Drag Coefficient Calculation

**Figure 14:**
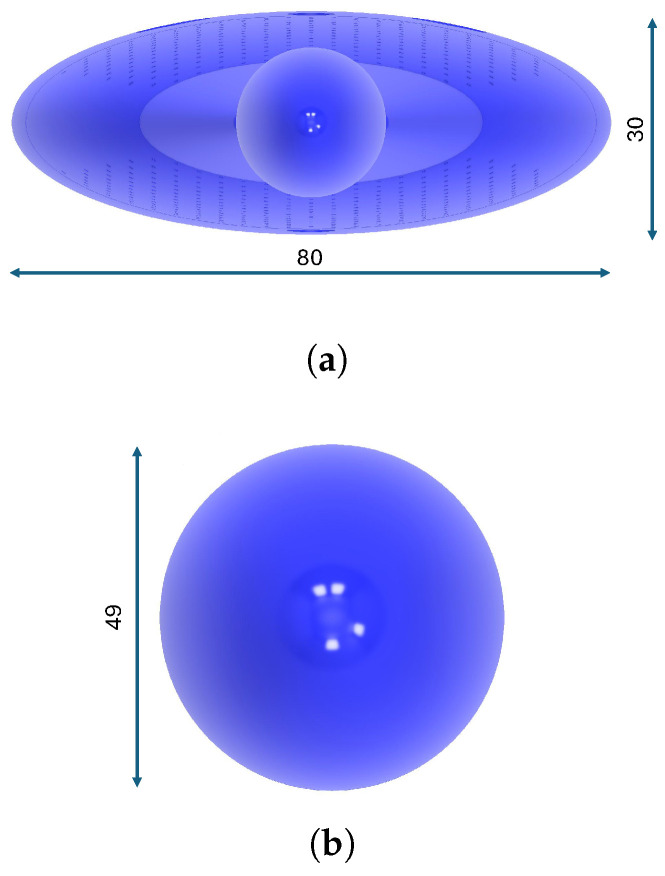
Highest cross-sectional areas perpendicular to the flow for the two prototypes. (**a**) The cross-sectional area of the penguin vessel. (**b**) The cross-sectional area of the torpedo vessel. All the dimensions are in millimeters.

The next quantity of interest is the force, measured as described in the previous sections. The experiments were repeated for all test types, with and without bubbles in both vessels, at low and high pressure. The force results for each case were then averaged across all the tests with the same conditions. Equation (7) is used to compute the drag coefficient $c_{d}$:

(7) $$c_{d}=\frac{2\bar{F}}{\rho {\bar{v}}^{2}A}$$

where *F* is the measured drag force, ρ is the density of the fluid, *v* is the flow velocity relative to the vessel and *A* is taken as the highest cross-sectional area perpendicular to the flow. The cross-sectional area *A* is identical for both vessels, with a value of 1885$\;{\mathrm{mm}}^{2}$, as depicted in Figure 14. Therefore, $c_{d}$ can be used as a relative metric to compare the different air-injection conditions, rather than as a steady-state hydrodynamic drag coefficient.

For the calculation of $c_{d}$, the average vessel velocity was utilized, as velocity measurements were limited to a small segment of the test using the camera and computer vision algorithm. The drag force used is the average force measured in the same time interval.

#### 4.2.3. Bubble Dimension Tracking

**Figure 15:**
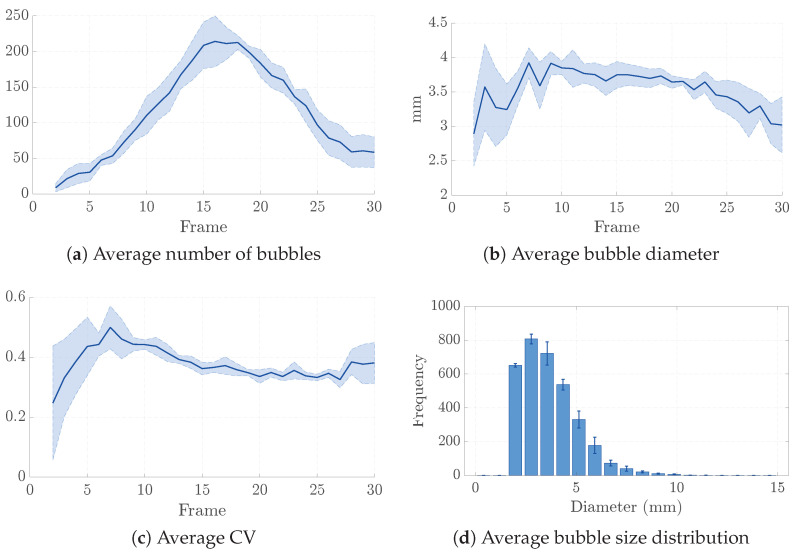
Bubble analysis for low pressure (0.5 bar, 0.41 L/s) in the penguin vessel. The shaded area and error bars correspond to ±σ.

**Figure 16:**
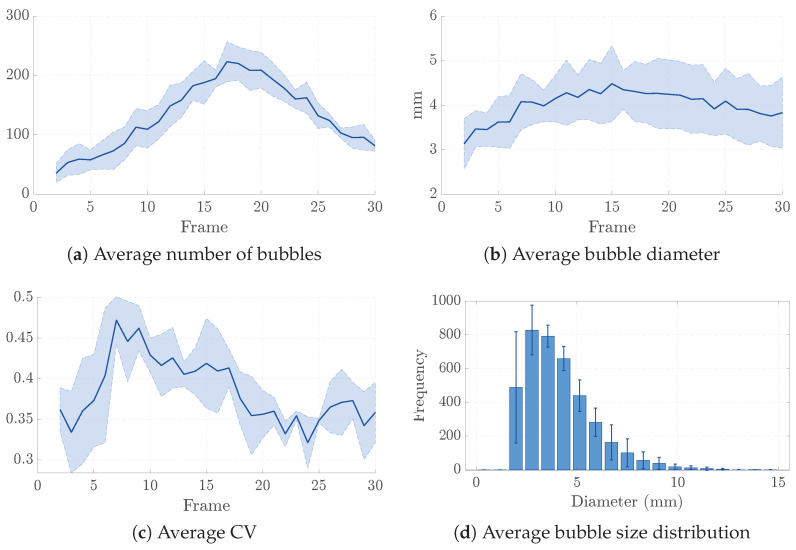
Bubble analysis for high pressure (1.5 bar, 0.59 L/s) in the penguin vessel. The shaded area and error bars correspond to ±σ.

**Figure 17:**
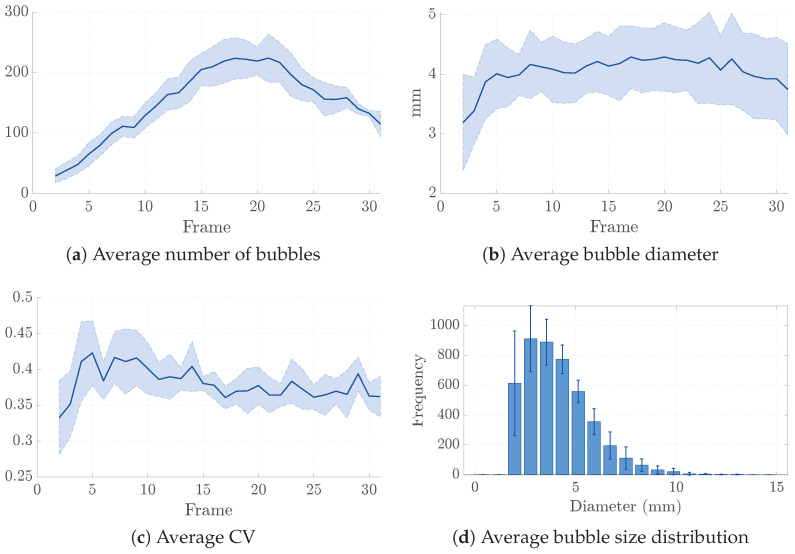
Bubble analysis for low pressure (0.5 bar, 0.41 L/s). in the torpedo vessel.

**Figure 18:**
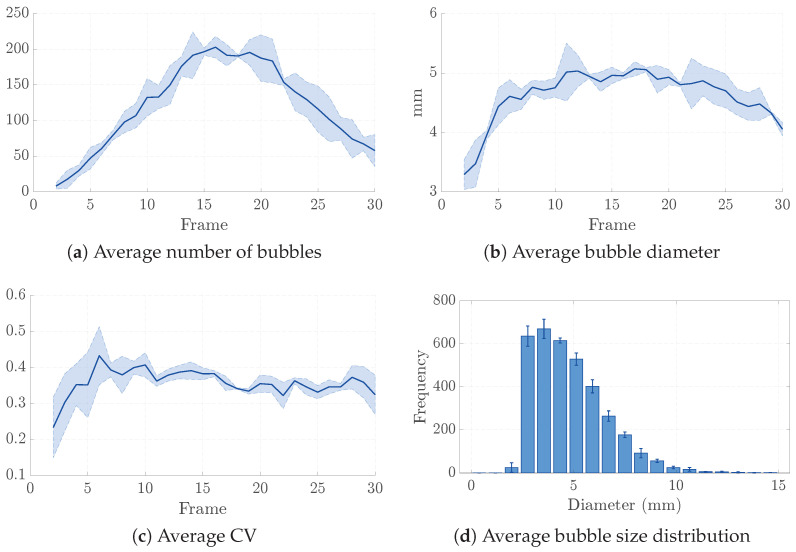
Bubble analysis for high pressure (1.5 bar, 0.59 L/s) in the torpedo vessel. The shaded area and error bars correspond to ±σ.

During the tests, when the air supply was open, a side-view camera captured the motion of the ejected bubbles. The recorded videos were subsequently processed by the computer vision code explained in Section 4.1.3, obtaining the graphs shown in Figure 15, Figure 16, Figure 17 and Figure 18. The results reported in the figures show the average number of bubbles and their diameter, together with the coefficient of variation CV and the distribution of observed bubble sizes. The variation in the average number of bubbles in Figure 15a, Figure 16a, Figure 17a and Figure 18a does not depend on a change in experimental conditions, but on the fact that the portion of the vessel captured by the camera varies over time. All the measurements were performed in steady-state conditions, once the vessels had reached a constant velocity.

Figure 15 and Figure 16 present the summary of bubble tracking for both high- and low-pressure conditions using the penguin-shaped prototype. In addition to the average bubble count, the computer vision algorithm also reports the total number of air bubbles identified during each test. At high pressure, the total bubble count is 3900, while at low pressure, it decreases to 3376. This outcome is expected, as higher pressure causes more air to exit the vessel’s surface, resulting in increased bubble formation. The graphs further indicate that at 1.5 bar, the greater number of bubbles is accompanied by larger bubble dimensions. Larger, coalescing bubbles are less effective at remaining near the vessel wall; instead of forming a stable air-rich layer, they tend to detach, migrate away from the body, and increase flow disturbances, thereby diminishing or reversing the drag-reduction effect. In these experiments, material and manufacturing limitations resulted in average bubble diameters of ranging between 3.5 mm and 4 mm, which are significantly larger than those produced by penguins, typically a few micrometers in diameter. Additionally, the bubbles exited the vessel at higher velocities than observed in the natural phenomenon, leading to reduced attachment to the vessel surface.

In Figure 17 the low-pressure tests for the torpedo vessel appear to generate more bubbles with respect to the other cases; however, this is primarily because, at higher pressure, bubbles merge rapidly into larger ones before being detected by the algorithm. Examination of the size distribution diagrams for both pressure conditions reveals that, at higher pressure, smaller bubbles (approximately 2 mm in diameter) are nearly absent, whereas they constitute a significant portion of the distribution at lower pressure. This phenomenon contributes to the observed drag coefficient results, as larger bubbles increase the surface area and do not contribute to drag reduction.

**Figure 19:**
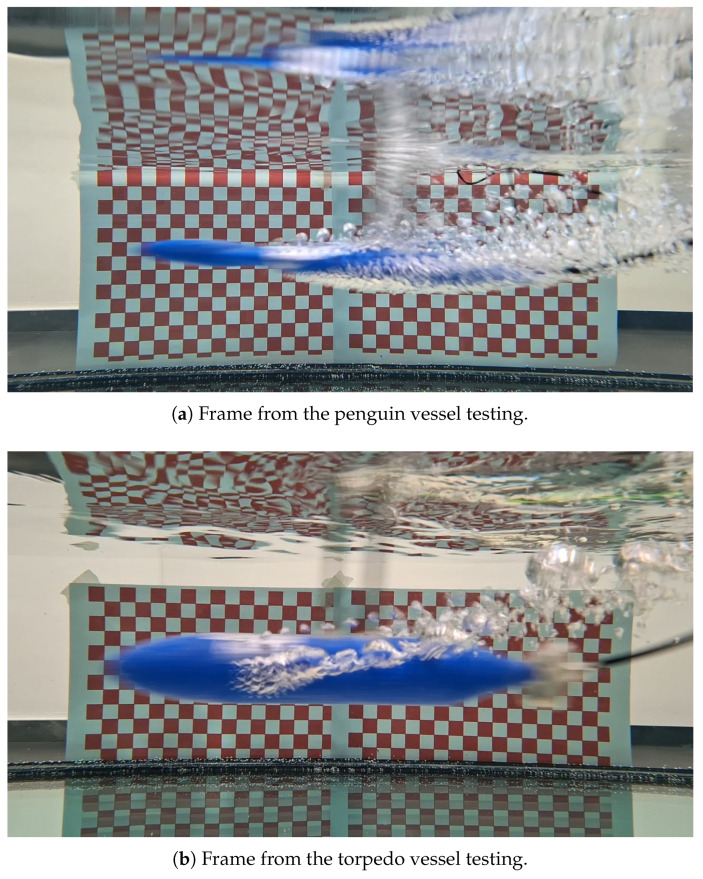
Frames showing the side view of bubble attachment during tests with the two prototypes.

**Figure 20:**
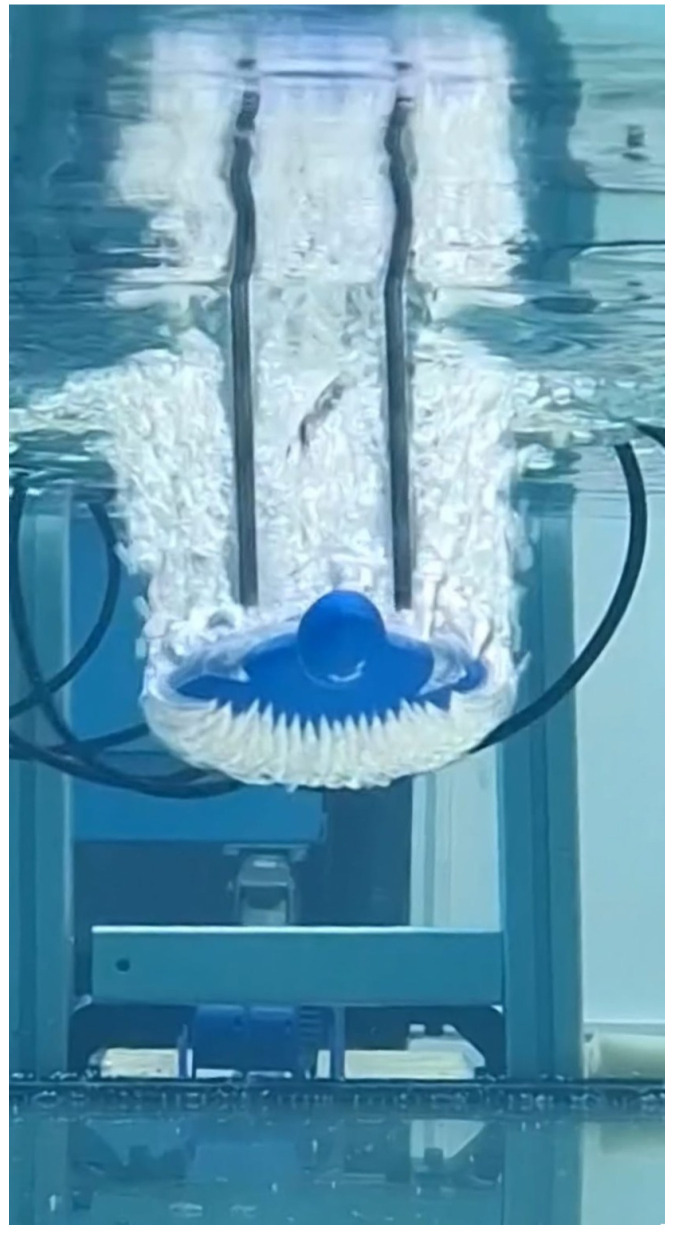
Frame of the front view of the penguin vessel while moving.

Figure 19 illustrates the differences in bubble attachment between the penguin and torpedo vessels. For the penguin vessel, the entire body is covered by bubbles, whereas for the torpedo vessel, most air exits through the sides, following the path of least resistance. Consequently, the bubble coating does not adhere to the torpedo body. Figure 20 depicts the formation of the bubble film beneath the penguin vessel.

Figure 20 shows the front view of the penguin vessel moving during an experiment with bubble injection. The air injection holes are visible, producing a blanket of air bubbles surrounding the vessel.

A summary of the experimental results is provided in Table 3. The force column reports the value of the averaged force trace, averaged over the period of time where steady-state conditions are achieved. The drag coefficient $c_{d}$ was computed using the mean force over the same time interval used for velocity estimation.

#### 4.2.4. Bubble Coverage

This section reports the bubble coverage ratio (BCR), which is computed in order to analyze the effectiveness of the bubble ejection system developed in this study. Air lubrication depends mostly on the ability of the air ejection system to surround the underwater body with air bubbles. Thus, the underwater vessel moves in a biphasic fluid, where bubbles contribute to the reduction in hydrodynamic resistance and total drag force.

Since the available recordings were acquired exclusively from a lateral viewpoint, the BCR is evaluated using the projected side view of the underwater vessels. Although the available data does not represent a real 3D reconstruction of the prototype surrounded by air bubbles, the projected lateral-view frames can be used to compute the ratio of the surface area covered by bubbles during the tests.

The BCR is defined as the fraction of the projected visible hull area occupied by bubbles. For each video frame, it is computed as:

$$BCR=\frac{A_{bubble}^{proj}}{A_{ROI}^{proj}}$$

where $A_{bubble}^{proj}$ is the projected area occupied by bubbles and $A_{ROI}^{proj}$ is the projected area of the visible hull that belongs to the region of interest. The value of BCR approaches 100% as a higher portion of the hull area is covered by bubbles, while it decreases if bubbles do not sufficiently cover the region of interest.

The area of the region of interest on the hull is first determined by projecting the lateral view of the CAD models of each prototype. The experimental videos are then processed using the bubble tracking code. The value of $A_{bubble}^{proj}$ is computed as the time-averaged bubble area within the region of interest during the period when bubble ejection is active. Table 4 summarizes the BCR obtained for each test condition. For both prototypes, the average BCR is higher under low air pressure (0.5 bar) than under high air pressure (1.5 bar). The results for 1.5 bar show higher variability, probably due to more chaotic bubble motion caused by the higher pressure. Although these results are derived from a two-dimensional analysis, they suggest that low-pressure conditions are preferable to high-pressure ones, as they produce wider bubble coverage around the prototype’s hull.

## 5. Discussion

Two prototypes with distinct shapes were employed to evaluate the drag-reduction capability of an air lubrication system. The distribution of air bubbles on the outer surfaces of the prototypes was found to depend on body shape. The torpedo-shaped body did not exhibit drag reduction with air lubrication, likely because its cylindrical form retains fewer bubbles on its underside compared to the penguin-shaped body. The penguin-shaped vessel, featuring an elliptical cross-section, retained more air bubbles during underwater motion, which enhanced drag reduction. This effect can be attributed to the elliptical cross-section’s shallower underside angle, which slows the ascent of bubbles as they exit the vessel.

The correlation between drag reduction and bubble ascent velocity is a possible explanation why increasing flow rate and pressure does not benefit the vessel. In fact, when bubbles are released at higher velocity, they struggle to remain attached to the outer surface of the prototype, thus failing to reduce the shear stress on the vessel’s outer surface. At elevated pressures, the increased number of bubbles promotes coalescence into larger bubbles. This effect was particularly pronounced during torpedo-shaped vessel testing and led to increased drag at higher pressure and flow rates.

Comparison with the biological system of the penguin indicates that injected bubbles can reduce drag in the penguin-inspired prototype under low-pressure conditions. The contrast with the torpedo-shaped body demonstrates that body geometry significantly influences bubble retention and, consequently, the effectiveness of bubble-assisted drag reduction. The experimental results suggest that adopting a body shape that allows more air bubbles to be retained around the body can reduce the total drag during underwater motion.

Although the flow regime achieved with the two prototypes resembles that observed in Adélie penguins, the results permit only a partial comparison with the natural phenomenon, as they cannot be generalized to larger penguin species such as the emperor penguin. Additionally, the ability to control bubble size by adjusting air pressure and flow rate is constrained by the minimum hole diameter achievable with 3D printing. Consequently, the bubbles generated in this study are larger than the ones released by real-life penguins, which are expected to be much smaller because of the fine porous structure of the plumage. This fact limits the effectiveness of replicating the natural phenomenon. Furthermore, the comparison between the two prototypes only partially isolates the effect of overall morphology, since both bodies share the same length and maximum frontal area but differ in wet surface area and volume.

## 6. Conclusions

This study conducted a preliminary experimental investigation of a penguin-inspired air-lubrication concept for underwater bodies. Two 3D-printed prototypes, a penguin-inspired body and a torpedo-shaped reference body, were tested in a dedicated towing tank under identical air-supply conditions. The results demonstrate that the effect of bubble injection is highly dependent on body geometry. Under low-pressure air injection, the penguin-inspired body exhibited a 31.5% reduction in drag coefficient compared to the condition without bubbles. Under identical conditions, the torpedo-shaped body did not experience drag reduction. At higher air pressures, both prototypes showed degraded performance, consistent with the formation of larger, more coalescent bubbles and reduced near-wall retention.

Video-based bubble analysis revealed that the penguin-inspired geometry facilitated greater bubble coverage along the lower surface, while the torpedo-shaped body permitted a larger fraction of bubbles to detach laterally. These findings support the hypothesis that bubble-assisted drag reduction is influenced not only by air injection but also by the body’s morphology in maintaining an air-rich region near the surface.

Even though the current prototype does not represent a dynamically scaled replica of penguin plumage, as the generated bubbles are millimetric, the air supply is external, and the surface does not replicate the deformable porous structure of feathers, the results demonstrate that a simplified bioinspired morphology can enhance bubble retention and drag response compared to a conventional axisymmetric reference body. Future research should focus on improved control of bubble size, a possible viable solution is via microporous media and fluidic oscillation, and testing across a broader range of Reynolds numbers and air-flow rates. A further development would involve internalizing the air supply system to obtain a more compact vessel design. Specifically, integrating a pressurized air tank within the vessel’s body would eliminate the need for an external air feed line, allowing for more precise control of the outlet pressure. Beyond improving compactness and control accuracy, this design modification would represent an important step toward enabling real-world deployment of the system.

## Figures and Tables

**Table 1:** Biological inspiration and engineering translation adopted in this study.

Biological Feature	Engineering Translation	Simplification
Dense, porous feather-down assembly capable of retaining air	Internal air chamber and porous diffuser used to distribute air before release	Feather microstructure and deformability are not reproduced
Fine wire-like network of barbs, barbules, and hamuli	Porous diffuser and external release holes used to generate a bubble cloud	Generated bubbles are larger than the fine bubbles expected from natural plumage
Pressure-dependent release of air during ascent	Controlled air injection through 0.6 mm holes near the upstream region of the body	Air release is imposed by supply pressure rather than produced by hydrostatic decompression and feather motion
Air-rich region close to the body surface	Bubble cloud intended to remain near the surface	Near-wall coverage depends on geometry, injection pressure, and bubble coalescence
Possible active management of plumage through preening and feather orientation	Fixed 3D-printed body with constant geometry during each test	No active surface control or feather-like motion is implemented.

**Table 2:** Comparative data table for the prototypes.

Data	Penguin-Inspired Vessel	Torpedo-Inspired Vessel
Length	293 mm	293 mm
Max frontal area	1885 ${\mathrm{mm}}^{2}$	1885 ${\mathrm{mm}}^{2}$
Surface area in contact with water	35,880 ${\mathrm{mm}}^{2}$	40,604 ${\mathrm{mm}}^{2}$
Volume	139,571 ${\mathrm{mm}}^{3}$	242,000 ${\mathrm{mm}}^{3}$
Mass	92.07 g	123.84 g
Number of holes	600	600
Hole diameter	0.6 mm	0.6 mm
Air injection distance from the head	102.5 mm	84.3 mm
Print layer height	0.1 mm	0.1 mm

**Table 3:** Summary of the measured drag response for the two prototypes. Values are reported as apparent drag coefficients for relative comparison between test conditions.

Body Shape	Air Pressure	Air Flow Rate	Average Force	$c_{d}$	Change vs. No Air
Penguin	No air	0 L/s	2.05 ±0.53 N	0.89	–
Penguin	0.5 bar	0.41 L/s	1.62 ±0.45 N	0.61	−31.5%
Penguin	1.5 bar	0.59 L/s	2.08 ±0.55 N	0.77	−13.5%
Torpedo	No air	0 L/s	2.44 ±0.75 N	1.43	–
Torpedo	0.5 bar	0.41 L/s	2.33 ±0.72 N	1.87	+30.8%
Torpedo	1.5 bar	0.59 L/s	3.52 ±0.91 N	2.67	+86.7%

**Table 4:** Summary of the bubble coverage analysis on the two prototypes in different testing conditions.

Body Shape	Air Pressure	Air Flow Rate	Hull Projected Area	BCR
Penguin	No air	0 L/s	$12,940\;{\mathrm{mm}}^{2}$	–
Penguin	0.5 bar	0.41 L/s	$12,940\;{\mathrm{mm}}^{2}$	48.70±2.15%
Penguin	1.5 bar	0.59 L/s	$12,940\;{\mathrm{mm}}^{2}$	46.53±5.70%
Torpedo	No air	0 L/s	$16,350\;{\mathrm{mm}}^{2}$	–
Torpedo	0.5 bar	0.41 L/s	$16,350\;{\mathrm{mm}}^{2}$	37.57±3.08%
Torpedo	1.5 bar	0.59 L/s	$16,350\;{\mathrm{mm}}^{2}$	33.11±6.30%

## Data Availability

The data and the processing scripts supporting the findings of this study are available from the corresponding author upon reasonable request. Video files are available as Supplementary Materials.

## Supplementary Materials

The following supporting information can be downloaded at https://www.mdpi.com/article/10.3390/biomimetics11070501/s1. Video S1a–e: tests for the penguin vessel at high pressure; Video S2a–e: tests for the penguin vessel at low pressure; Video S3a–e: tests for the penguin vessel without air ejection; Video S4a–e: tests for the torpedo vessel at high pressure; Video S5a–e: tests for the torpedo vessel at low pressure; Video S6a–e: tests for the torpedo vessel without air ejection.
